# Glyburide and retinoic acid synergize to promote wound healing by anti-inflammation and RIP140 degradation

**DOI:** 10.1038/s41598-017-18785-x

**Published:** 2018-01-16

**Authors:** Yi-Wei Lin, Pu-Ste Liu, Kasey Ah Pook, Li-Na Wei

**Affiliations:** Department of Pharmacology, University of Minnesota Medical School, Minneapolis, Minnesota 55455 USA

## Abstract

Chronic inflammation underlies the development of metabolic diseases and individuals with metabolic disease often also suffer from delayed wound healing due to prolonged inflammation. Resolving inflammation provides a therapeutic strategy in treating metabolic diseases. We previously showed that during an anti-inflammatory response when macrophages were alternatively (M2) polarized, retinoic acid (RA) dramatically activated arginase 1 gene (*Arg1*), a gene crucial for wound healing. Here we report that a widely used sulfonylurea drug for type 2 diabetes mellitus (T2DM), glyburide, enhances the anti-inflammatory response and synergizes with RA to promote wound healing. Our data also delineate the mechanism underlying glyburide’s anti-inflammatory effect, which is to stimulate the degradation of a pro-inflammatory regulator, Receptor Interacting Protein 140 (RIP140), by activating Ca2+/calmodulin-dependent protein kinase II (CamKII) that triggers specific ubiquitination of RIP140 for degradation. By stimulating RIP140 degradation, glyburide enhances M2 polarization and anti-inflammation. Using a high-fat diet induced obesity mouse model to monitor wound healing effects, we provide a proof-of-concept for a therapeutic strategy that combining glyburide and RA can significantly improve wound healing. Mechanistically, this study uncovers a new mechanism of action of glyburide and a new pathway modulating RIP140 protein degradation that is mediated by CamKII signaling.

## Introduction

Metabolic diseases such as Type II diabetes mellitus (T2DM) cast a huge burden on global health. These patients exhibit multiple symptoms such as hyperglycemia, insulin resistance, hypertension and dyslipidemia^1^, as well as debilitating, delayed wound healing^2,3^. One common underlying condition of metabolic diseases is systemic inflammation^4^; as such, resolving inflammation is an important goal in managing these symptoms^5^. This is especially critical to aid patients of metabolic diseases in wound recovery.

Retinoic acid (RA) exerts pleiotropic effects and is widely used to treat various health conditions particularly those involving the immune system^6,7^. In a clinical setting, retinoids have been used to treat cancers such as acute promyelocytic leukemia, although systemic toxicity has caused wide concerns^8,9^. Additionally, there is a long history of topical application of RA in dermatology, such as to enhance wound healing, treat acne, and reverse skin aging caused by UV damage. These topical applications have proven relatively safe^10–13^. Recently, we have found that in macrophage polarization, a process essential to innate immunity, RA treatment significantly elevates the expression of Arginase 1 (Arg1), a gene product critical to wound healing process. This happens particularly in the phase of alternative (M2) macrophage polarization, and therefore, it can potentially boost the wound healing process^14^.

The management of metabolic diseases especially T2DM and gestational diabetes mellitus includes drugs that stimulate insulin secretion, such as glyburide (also known as glibenclamide)^15,16^, a drug belonged to the sulfonylurea class. Glyburide can also inhibit sulfonylurea receptor 1-transient receptor potential melastatin 4 (Sur1-Trpm4) channels to protect patients from ischemic and hemorrhagic strokes^17^. It can also inhibit Cryopyrin/Nalp3 inflammasome pathway^18^. Additionally, our unpublished preliminary data suggest an anti-inflammatory potential for glyburide. Given that RA can boost Arg1 expression, and that glyburide is potentially anti-inflammatory, we propose that combining RA and glyburide may be synergistically beneficial to the management of wounds, especially for patients suffering from, or in the process of developing, metabolic diseases. The study is to test this therapeutic strategy for wound healing in a high fat diet (HFD)-induced obesity mouse model that mimics the condition of chronic inflammation.

Our data show that glyburide and RA indeed synergize to facilitate wound healing in these animals. We also uncover a new mechanism of action of glyburide, which is by stimulating protein degradation of a key inflammatory coregulator named nuclear receptor coregulator RIP140 (Nrip1). RIP140 is a wide spectrum transcription co-regulator^19,20^. For innate immune cells especially macrophages, RIP140 is pro-inflammatory because it is a cofactor of NF-kB, facilitating M1 polarization^21^, and an inhibitor of STAT6, suppressing M2 polarization^22^. Therefore, silencing RIP140 expression or reducing its protein levels in macrophages generally leads to M2 polarization (anti-inflammation). Since RIP140 gene (*Nrip1*) expression is maintained largely constant in macrophages^23,24^, its protein level is primarily regulated by post-transcriptional control. We have previously determined that RIP140 can be degraded by Syk-mediated tyrosine phosphorylation on Tyr364, Tyr418 and Tyr436 in the pathological context of LPS-induced inflammation, which prevents septic shock^21^.

While the ability to control RIP140 protein level is highly desirable, especially for treating diseases related to, or caused by, inflammation, this strategy has remained a challenge due to the lack of therapeutic agents that could trigger effective and specific protein degradation of RIP140. The current study aims to identify potential therapeutic agents that are safe and can modulate RIP140 protein levels in order to promote resolution of a chronic inflammation-related condition. This effort has identified glyburide as a potential therapeutic agent. Given RA’s effect in elevating Arg1^14^, which is beneficial to wound healing, combining glyburide and RA would be predicted to synergistically facilitate the healing process of chronic wounds associated with inflammation. By examining signaling pathways, we also determine the mechanism of glyburide’s action in regulating RIP140′s protein level, which is mediated by CamKII signaling. This also represents a new regulatory pathway for protein quality control of RIP140.

## Results

### Glyburide improves wound healing in HFD-induced obese mice

**Figure 1:**
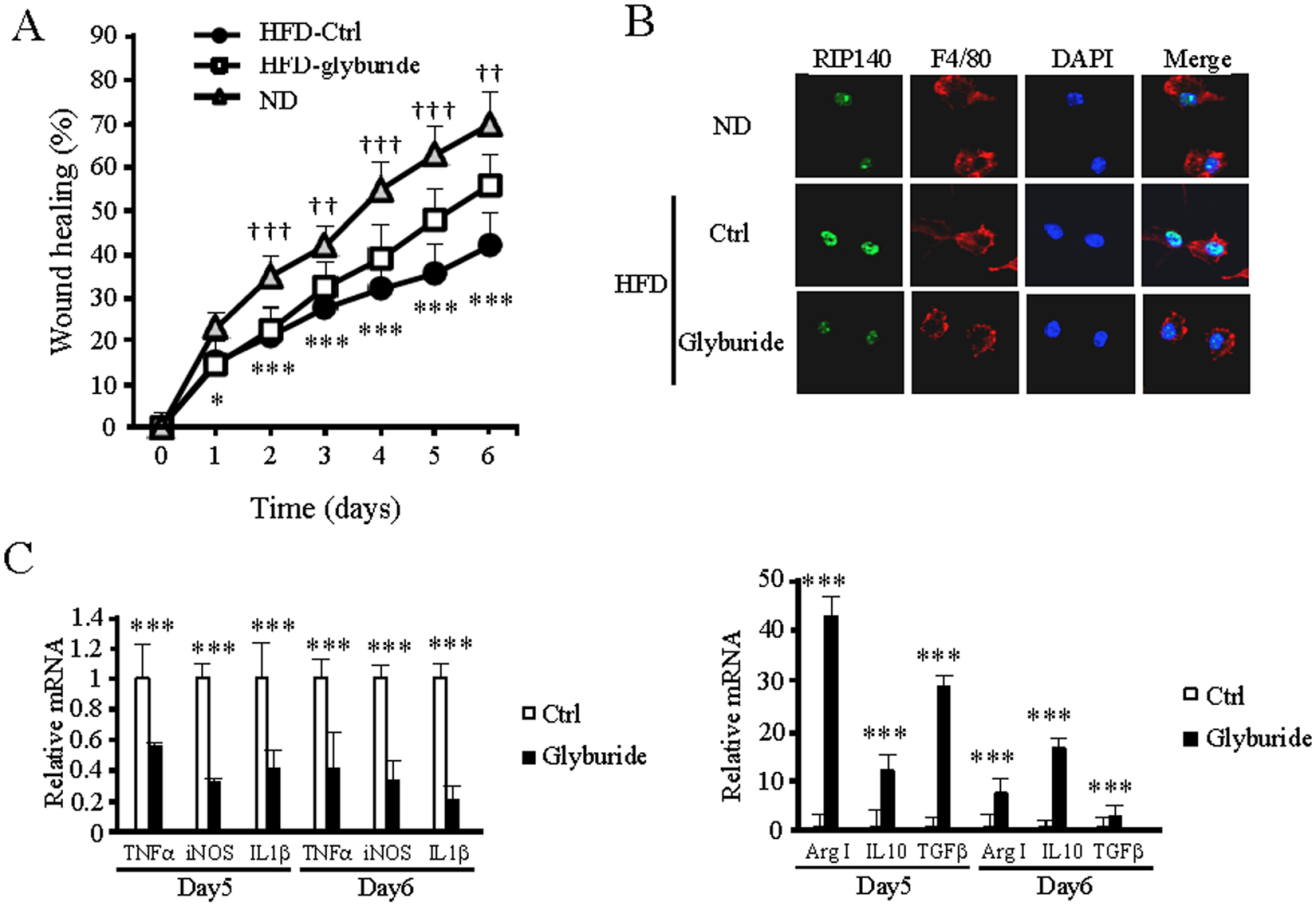
Glyburide improves wound healing in HFD-induced obese mice. (**A**) Daily record of wound closure in mice fed with ND, HFD, and HFD and treated with a control solvent DMSO (ND and HFD-Ctrl) or glyburide (HFD-glyburide). Data were presented as mean ± SD. A two-way ANOVA test was used *p < 0.05, **p < 0.01; ***p < 0.001. (ND vs. HFD-Ctrl); ^†^p < 0.05, ^††^p < 0.01, ^†††^p < 0.001 (HFD-Ctrl vs. HFD-glyburide) (N = 6 in each group), Ctrl = Control. (**B**) Immunofluorescence images of RIP140 in primary mouse macrophages isolated from wound. (**C**) qPCR analyses of M1 and M2 markers detected in wound tissues from HFD-Ctrl or HFD-glyburide animals. Student test was used and data were presented as means ± SD. ***p < 0.001 (N = 6 in each group).

We first determined the effects of glyburide in healing the wounds created in HFD-induced obesity mice. The results show that topical treatment of these mice with glyburide significantly improved their wound healing as compared to the control (Fig. 1A). We then examined if glyburide could alter RIP140 protein levels in the macrophage populations of wounded tissues. Indeed, glyburide treatment down-regulated RIP140 protein levels in macrophages collected from these wounds (Figs 1B and S1A, S1B). Further, glyburide treatment elevated their M2 (anti-inflammatory) markers and reduced their M1 (inflammatory) markers (Fig. 1C), confirming that glyburide is anti-inflammatory and can reduce RIP140 protein level in macrophages.

### Combining glyburide and RA enhances wound healing

**Figure 2:**
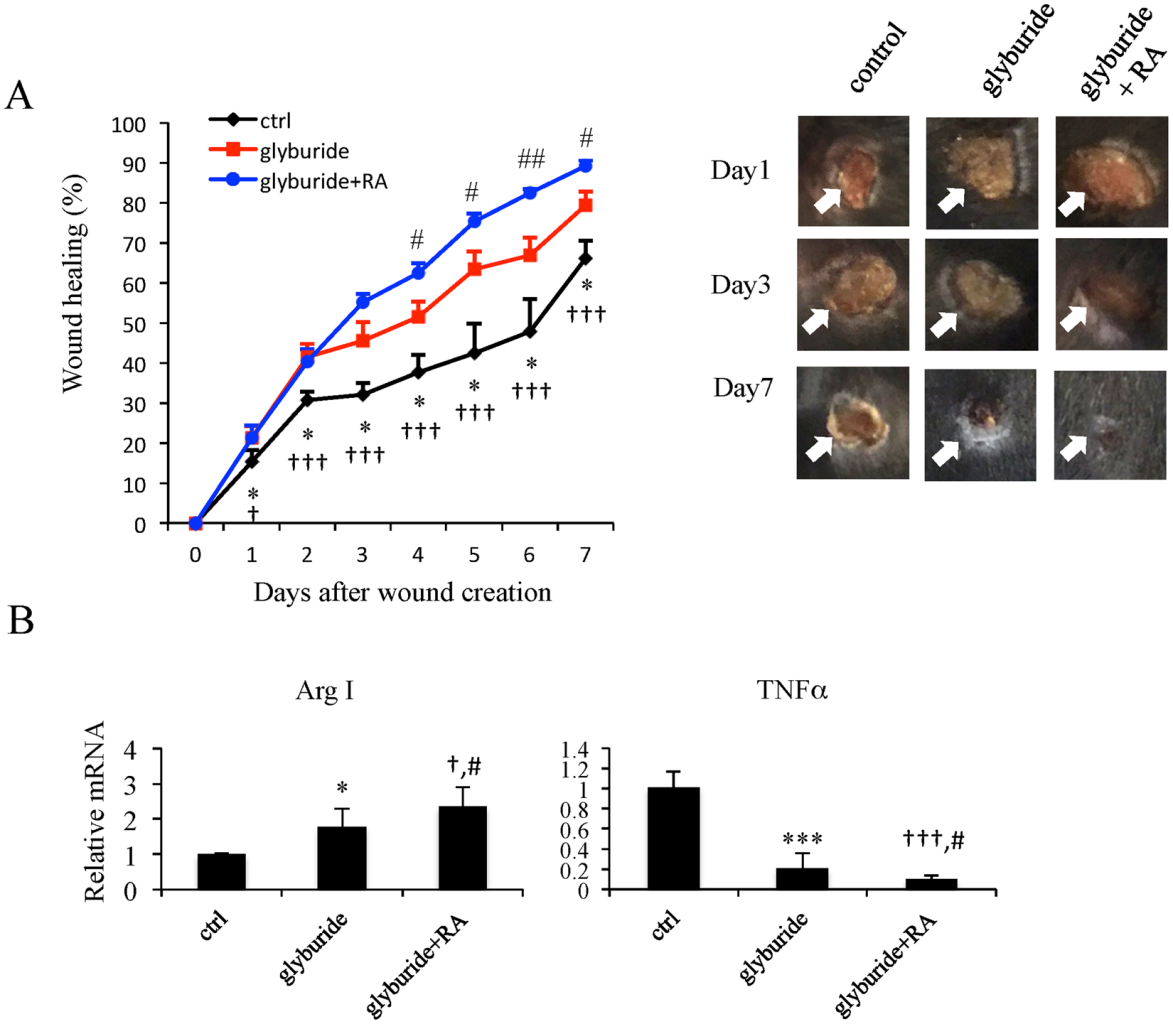
Co-treatment with glyburide and RA improves wound healing as compared to treatment of glyburide alone. (**A**) Left: Daily record of wound closure in HFD-mice treated with ctrl, glyburide, or glyburide/RA co-treatment. Data were presented as mean ± SD. A two-way ANOVA test was used *p < 0.05 (Ctrl vs. glyburide); ^†^p < 0.05, ^††^p < 0.01, ^†††^p < 0.001 (Ctrl vs. glyburide + RA); ^#^p < 0.05, ^##^p < 0.01 (glyburide vs. glyburide + RA) (N = 6 in each group), Ctrl = Control. Right: Representative cutaneous wound on day 1, 3 and 7 after wound creation. (**B**) qPCR analyses of M1 and M2 markers in wound tissues was presented as mean ± SD. A two-way ANOVA test was used *p < 0.05, ***p < 0.001 (Ctrl vs. glyburide); ^†^p < 0.05, ^†††^p < 0.001 (Ctrl vs. glyburide + RA); ^#^p < 0.05 (glyburide vs. glyburide + RA) (N = 6 in each group).

Our recent studies demonstrated that RA treatment in macrophage cultures or mice enhanced *Arg1* gene expression, which also improved wound healing^14^. Given anti-inflammatory effects of glyburide and Arg1-elevating activity of RA, we suspected that combining RA and glyburide might be able to further improve wound healing in a condition of chronic inflammation such as a HFD-induced obese condition. To test this possibility, we employed the same cutaneous wound model in three cohorts of obese mice all treated with HFD for 8-weeks. These include a control group (treated with a control cream), a group treated with daily topical glyburide application, and a group treated with topical glyburide for 2 days followed by the combination of glyburide and 0.1% tretinoin cream (RA) daily. Closure of the wounds was then monitored. As shown in Fig. 2A, the group treated with the combination of glyburide and RA significantly improved their wound healing as compared to the control and the group treated with glyburide alone (Fig. 2A). Consistently, gene expression analysis showed that combining RA and glyburide boosted anti-inflammation, indicated by an increase in the M2 marker and a decrease in the M1 marker in the wound tissues (Fig. 2B).

### Glyburide stimulates RIP140 protein degradation in macrophages

**Figure 3:**
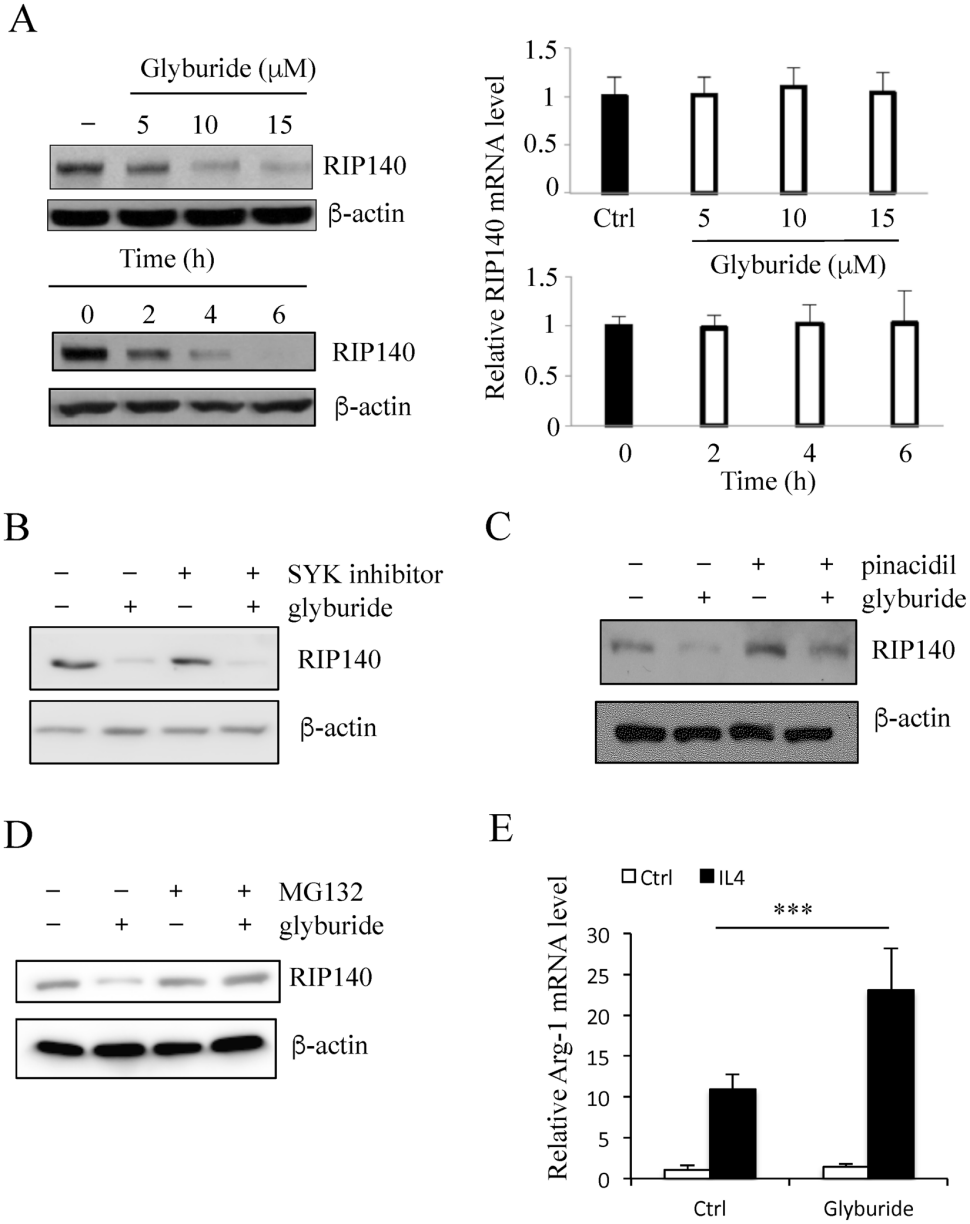
Glyburide stimulates RIP140 protein degradation in macrophage. (**A**) Western blot (left) and qPCR (right) analyses of RIP140 in mouse PMs treated with glyburide at three doses, and at three time intervals using 15 uM of glyburide. (**B**) Western blot of RIP140. The SYK inhibitor failed to block the effect of glyburide in Raw 264.7 cells. (**C**) Western blot of RIP140, showing the rescue of glyburide stimulated RIP140 degradation with pinacidil treatment in Raw 264.7 cells. (**D**) Western blot of RIP140, showing the rescue of glyburide stimulated RIP140 degradation with MG132 treatment in Raw 264.7 cells. (**E**) qPCR analyses of Arg-1 in Raw 264.7 cells treated with glyburide. Student test was used. All experiments were performed three times and presented as mean ± SD; ***P < 0.001.

As shown in Fig. 1B, glyburide reduced RIP140 protein levels in macrophages collected from wound tissues. To determine the mechanism of glyburide’s action specifically in macrophages, we employed both primary mouse peritoneal macrophage (PM) and a mouse macrophage cell line Raw 264.7 for the studies. In PM cultures, glyburide reduced RIP140 protein levels in a dose- and time-dependent manner (Fig. 3A left panel); whereas RIP140 mRNA levels remained relatively constant (Fig. 3A right panel). This led us to suspect that glyburide could reduce RIP140 protein levels by triggering its degradation. We previously identified that, in the patho-physiological context of LPS-induced inflammation, RIP140 was degraded by Syk-stimulated Tyr phosphorylation on Tyr364, Tyr418, and Tyr436 that stimulated its ubiquitination and degradation^21^. We then determined whether glyburide-stimulated RIP140 protein down regulation was mediated by Syk-stimulated degradation. As shown in Fig. 3B, the effect of glyburide was not related to Syk-stimulated degradation because a Syk inhibitor failed to block the effect of glyburide. The mechanism of action of glyburide in pancreatic beta cells has been attributed to, primarily, its activity in inhibiting ATP-sensitive potassium channel (K_ATP_)^25^. However, a potassium channel opener, pinacidil, also failed to effectively prevent glyburide-induced RIP140 degradation (Fig. 3C), ruling out the effects through altering K_ATP_. Interestingly, a proteasome inhibitor MG132 could effectively prevent glyburide’s effect in down-regulating RIP140 protein level (Fig. 3D), suggesting that glyburide triggered RIP140 degradation via a proteasome-mediated degradation pathway that is different from Syk-stimulated Try phosphorylation on RIP140. As predicted, glyburide-treated macrophages were more prone to IL-4 stimulated M2 activation (for anti-inflammatory response) (Fig. 3E), because RIP140 level was reduced.

### Glyburide stimulates RIP140 degradation by activating CamKII that phosphorylates RIP140

**Figure 4:**
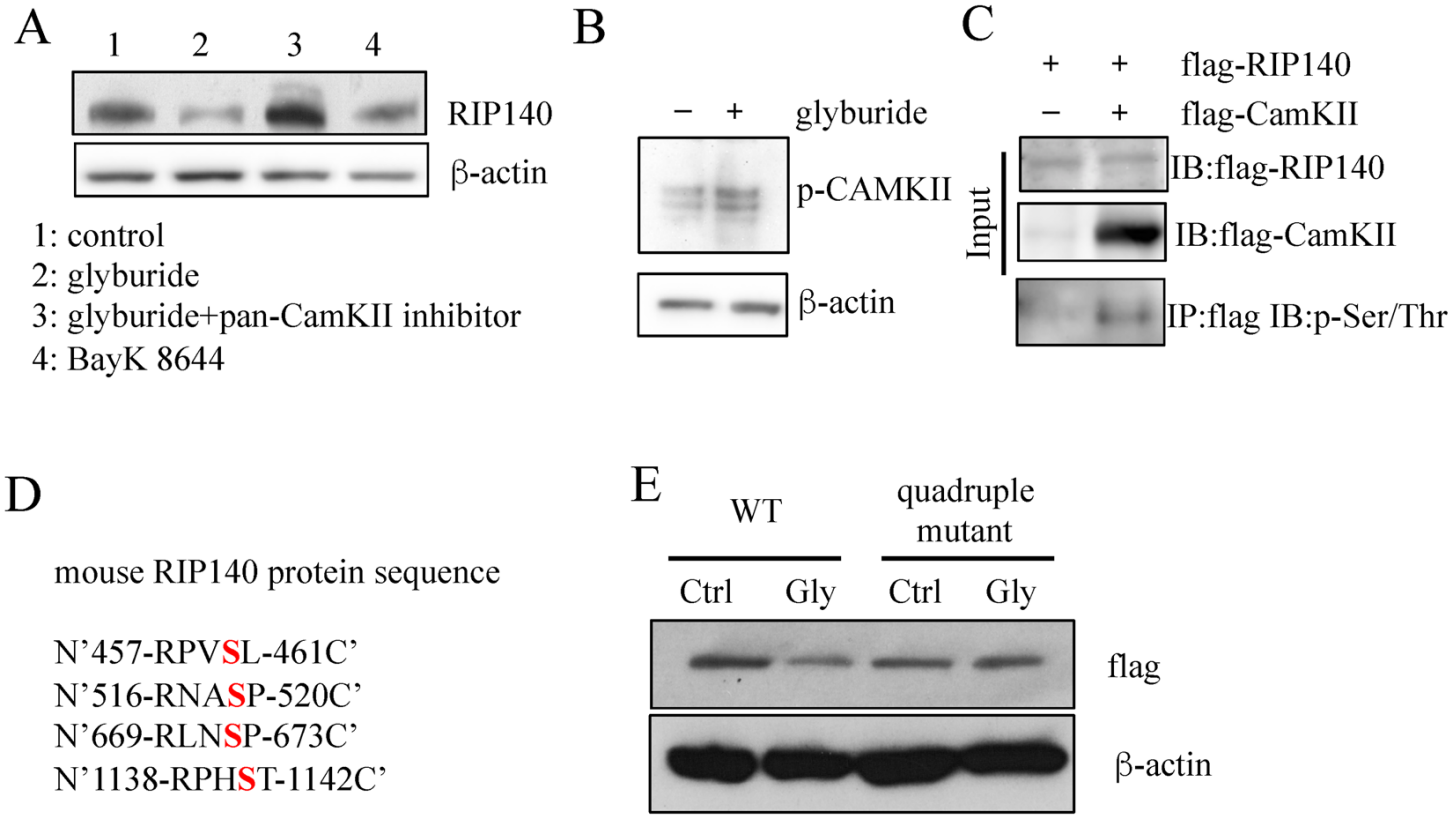
Glyburide stimulates RIP140 degradation through activating CamKII signaling pathway. (**A**) Western blot of RIP140, showing the effects of CamKII inhibitor and calcium channel activator in Raw 264.7 cells. (**B**) Western blot of phospho-CamKII demonstrating activation of CamKII in Raw 264.7 cells by glyburide treatment. (**C**) *In vitro* CamKII kinase assay showing CamKII-phosphorylation at serine residues of RIP140. (**D**) Predicted CamKII target sites on mouse RIP140. (**E**) Western blot of RIP140 showing glyburide-stimulated degradation of the wild type but not the quadruple mutant RIP140 in Raw 264.7 cells.

Glyburide is also known to elevate intracellular calcium concentration, we thus examined whether altering intracellular calcium concentration and/or calcium signaling in macrophage could affect its endogenous RIP140 protein level. The data (Fig. 4A) show that BayK8644, a calcium channel activator, induced RIP140 degradation, and a pan Ca2+/calmodulin-dependent protein kinase II (CamKII) inhibitor, KN-93, effectively blocked glyburide-induced RIP140 degradation. Further, glyburide indeed activated CamKII in this experimental system (Fig. 4B). In an *in vitro* CamKII assay, we found that CamKII could directly phosphorylate RIP140 (Fig. 4C). Based on the consensus sequence RXXS/TX of CamKII targets^26^, we predicted four possible CamKII target sites on RIP140 (Fig. 4C). We then generated a quadruple RIP140 mutant (S460A, S519A, S672A, S1141A, Fig. 4D) to eliminate CamKII substrate sites. This quadruple RIP140 mutant indeed was resistant to glyburide-induced degradation, confirming our prediction (Fig. 4E).

Taken together, these data show that glyburide is anti-inflammatory because it activates CamKII, which modifies RIP140 protein for proteosome-mediated degradation. Further, combining glyburide and RA would boost would healing because this strategy elevates Arg1 level in an enhanced anti-inflammatory condition. A proof-of-concept for this strategy is provided in our results showing more effective management of the wounds created in the HFD-induced obese condition.

## Discussion

This study provides a proof-of-concept for a new therapeutic regime in managing inflammation-associated chronic wounds, which is by combining two widely applied therapeutic agents, glyburide and RA. Glyburide promotes anti-inflammation, which sensitizes the system allowing more effective action of RA to elevate Arg1 level that is critical to wound healing. Both are widely applied therapeutics - glyburide is a relatively safe medication even for a long term use and tretinoin creams (RA) is safe when applied on the skin; therefore there should be little concern over toxicity when they are applied to manage topical wounds. The feasibility of developing this regime into an economical therapeutic to improve wound healing is quite high.

Calcium ion (Ca^2+^) plays a critical role in numerous physiological processes particularly in neuronal signal transmission, muscle contraction, and fertilization, etc.^27–29^. For the immune system, calcium signal contributes to the activation and differentiation of various immune cells including macrophages, monocytes, T cells, B cells, NK cells and dendritric cells^30,31^, etc. Study of *Drosophila* suggests that calcium acts as the earliest inflammatory signal to attract immune cell migration into the wound^32^. It is interesting that our current study shows that Ca^2+^ can also stimulate RIP140 protein degradation in macrophages, thereby contributing to anti-inflammation. RIP140 is an important regulator of inflammation; controlling its protein level is vital to the maintenance of immune homeostasis. The current study is the first to demonstrate protein quality control of RIP140 via calcium signaling.

Glyburide, as an anti-diabetic drug, is best known to stimulate insulin secretion in pancreatic beta cells. It has also been shown to be anti-inflammatory^18,33,34^, but the underlying mechanism was not clear. In the present study, we are able to delineate the mechanism of glyburide’s action in anti-inflammation, which is to facilitate RIP140 protein degradation through activating CamKII. This subsequently triggers posttranslational modification of RIP140 on Ser460, Ser519, Ser672, and Ser1141 and elicits its proteasome-mediated protein degradation. Interestingly, this degradation pathway is different from the degradation pathway triggered by LPS-induced inflammation where RIP140 is tyrosine phosphorylated on Tyr364, Tyr418 and Tyr436, which also leads to proteasome-mediated degradation. Conceivably, the patho-physiological context is crucial for RIP140 protein quality control, and it involves distinct signaling pathways in various physiological or pathological conditions to differentially modify RIP140. But all these lead to proteasome-mediated RIP140 protein degradation. To this end, it remains to be determined as to the physiological context where RIP140 may be degraded through endogenously activated CamKII.

RA, one of the active metabolites of retinoids, is important for a wide spectrum of biological processes and functions^35^. For innate immunity, RA can decrease pro-inflammatory cytokines production and increase anti-inflammatory cytokines secretion^7^. This is supported by epidemiological studies which demonstrate that RA deficiency alters immune responses to vaccines, infectious agents and auto-antigens, etc.^36^. In a clinical setting, all-trans-RA (tretinoin), 13-cis-RA (isotretinoin) and 9-cis-RA have all been shown to promote wound healing^37^ and applied in treating skin conditions. We have recently shown that RA enhances *Arg1* gene activation in IL4-stimulated M2, anti-inflammatory macrophages to facilitate wound healing^14^. The current study exploits this recent observation to develop a novel therapeutic regime for severe wounds such as those associated with chronic/systemic inflammation (using a HFD-induced obese mouse model). This regime should be safe for managing topical wounds; but its safety in systemic application for managing internal wounds remains to be further investigated.

RIP140 is a wide spectrum transcription co-regulator important for various biological processes^19,20^. Increasing evidences have revealed RIP140′s critical roles in regulating innate immunity. While numerous studies have all suggested that targeting RIP140 in macrophage can be a therapeutic strategy for inflammation-related diseases, as demonstrated using experimental systems like macrophage-specific knockdown^21,38^, bone marrow transplantation^39^, local injection of therapeutic macrophages^40^, and fecal microbiome transplantation^41^, a major challenge has to do with the lack of safe reagents/compounds that can be applied exogenously as a drug to stimulate RIP140 degradation. This current study is the first to identify such a pharmacological candidate that can facilitate RIP140 degradation to improve anti-inflammation. This information will be very helpful in future studies to screen for compounds that can modulate protein quality of RIP140 and the innate immune status. However, to provide more genetic evidence for this mechanism in the future, it is desirable to use genetically manipulated mouse models, such as mice carrying CamKII-resistant RIP140 mutation.

## Methods

### Reagents

Anti-RIP140 (Ab-42126) antibody was obtained from Abcam. Anti-b-actin, anti-CamKII, anti-phospho-CamKII, anti-mouse-IgG-HRP and anti-rabbit-IgG-HRP antibodies were purchased from Santa Cruz. Anti-phospho-Ser/Thr (9631 S) antibody was obtained from Cell Signaling. Anti-flag antibody, BayK 8644 (B112), glyburide (G2539) was from Sigma-Aldrich.

### Animals

All studies were performed using male C57Bl/6 mice purchased from The Jackson Laboratory. All experiments were approved by and in accordance with the guidelines and regulations of the University of Minnesota Institutional Animal Care and Use Committee. Animals were maintained in the animal facility of University of Minnesota on a 12 h light/dark photocycle. Mice were fed a normal diet (ND) (2018; Harlan Teklad, Madison, WI) or a high-fat diet (HFD) with 60% calories from fat (F3282; Bio-Serv, West Chester, PA).

### Cutaneous wound healing assay

Cutaneous wound healing assay was carried out as described^22^. 5-mm round-shape cutaneous wounds were made on shaved mice back using biopsy punch under anesthesia, to create 2 wounds per animal (n = 6). The reagents (glyburide, control cream and 0.1% tretinoin cream) were applied topically on wounds and wound size was recorded daily and analyzed by Image J.

### RNA Isolation and Gene Expression Analyses

Total RNA was isolated using TRIzol (Invitrogen) followed by manufacturer’s instruction. Reverse transcription and quantitative real-time PCR (qPCR) was performed as described previously using High-Capacity cDNA Reverse Transcription Kit containing RNase Inhibitor (Applied Biosystems) and Maxima SYBR Green qPCR Master Mixes (Thermo Scientific). Each gene-expression experiment was performed in triplicate and normalized to β-actin. Primers for Tnfα (QT00104006), IL1β (QT01048355), iNOS (QT00100275), IL10 (QT00106169), Arg1 (QT00134288) and TGFβ (QT00145250) were purchased from Qiagen.

### Flow Cytometry

Wound tissues were digested with 0.1% of collagenase and 0.01% of DNase I to disperse cells. Cell-surface antigens were blocked using Block (20 m g/mL; BD Biosciences). After blocking, cells were stained with antibodies or isotype control antibodies. Fluorophore-conjugated primary antibodies were purchased from BioLegend: F4/80-Alexa Fluor 647 (cat# 123122), and BD Bioscience: PE F(ab’)2 Donkey anti-Rabbit IgG (cat# 558416). Cells were analyzed on a BD Acuri C6 using FlowJo 10.0.6.

### ***In vitro*** CamKII assay

Flag-RIP140 and flag-CamKII fusion protein were made by TnT® Quick Coupled Transcription/Translation System (Promega) followed by manufacture’s instruction. Flag-RIP140 protein was incubated alone or with flag-CaMKII protein in 30 μl of *in vitro* kinase incubation buffer (25 mM HEPES/KOH, pH 7.4, 10 mM MgCl_2_, 1 mM CaCl_2_, 0.6 μM calmodulin, 6 μM ATP) at 30 °C for 10 minutes. The reaction was immunoprecipitated with anti-flag antibody and determined by western blotting using anti-phospho-Ser/Thr antibody.

### Cell culture

Primary peritoneal macrophages were isolated and maintained as described previously^39^. Raw 264.7 cells were maintained in DMEM medium as describe. Transfection was conducted using GenePorter 3000 (Genlatis) according to the manufacturer’s instructions.

### Plasmid Constructs

Point mutations involving residues Ser-460, Ser-520, Ser-672, and Ser1141 in mouse wild-type RIP140 (CMV/T7/FLAG) vector as template were made according to Q5 Site-Directed Mutagenesis Kit (NEB) according to the manufacturer’s instructions.

### Statistical Analysis

Experiments were carried out at least twice and presented as means ± SD. Unpaired two-tailed Student’s t test or two-way ANOVA was used for comparison between two groups. p values ≤ 0.05 were considered statistically significant (*p < 0.05; **p < 0.01; ***p < 0.001).

## Electronic supplementary material

Supplementary information
