# Supplementary material for: Glyburide and retinoic acid synergize to promote wound healing by anti-inflammation and RIP140 degradation
Source: Sci Rep. 2018 Jan 16;8:834. doi: 10.1038/s41598-017-18785-x (PMC5770422; doi:10.1038/s41598-017-18785-x)

Glyburide and retinoic acid synergize to promote wound healing by anti-inflammation and RIP140 degradation

Yi-Wei Lin, Pu-Ste Liu, Kasey Ah Pook and Li-Na Wei*

Department of Pharmacology, University of Minnesota Medical School, Minneapolis, Minnesota 55455, USA.

*** Correspondence:**
Li-Na Wei
612 Jackson Hall, 321 Church St. SE, Minneapolis, MN 55455
Fax number: 612-625-8408
Tel number: 612-625-9402
Email: weixx009@umn.edu

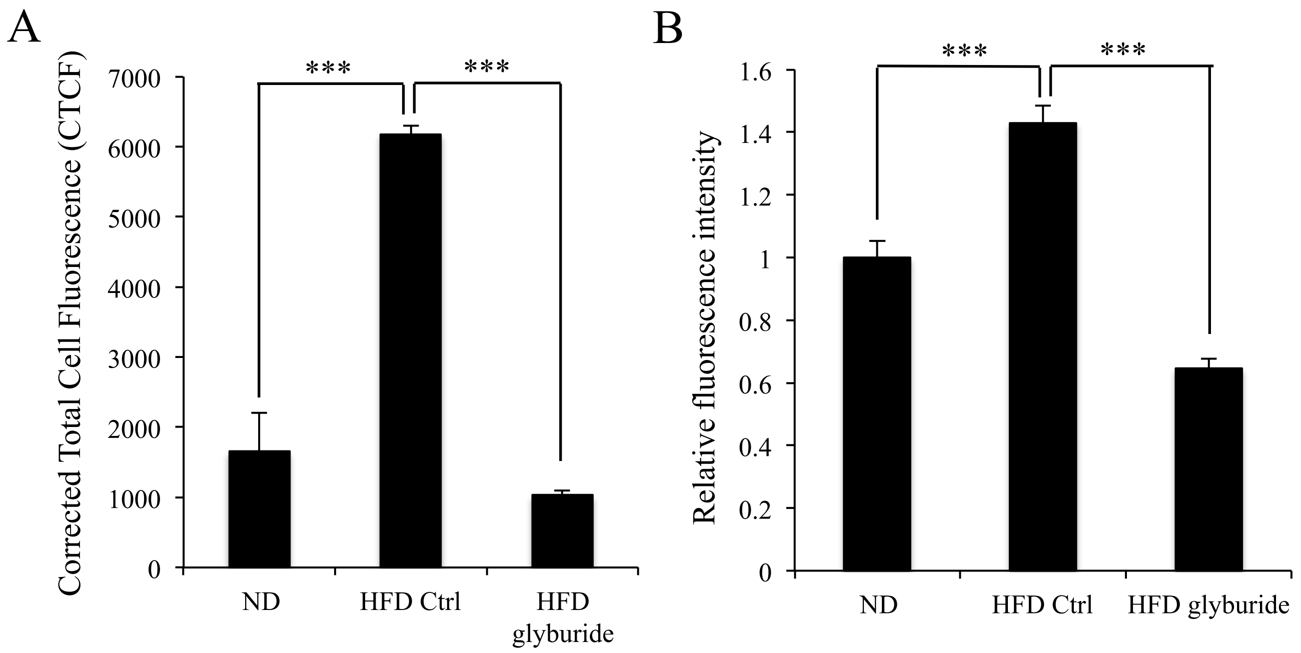

Fig. S1. Glyburide reduces RIP140 expression in macrophages collected from the wound tissues. A. Corrected total cell fluorescence (CTCF) of immunofluorescence of RIP140 protein in primary mouse macrophages isolated from the wounds. B. FACS analysis of RIP140 expression levels in primary mouse macrophages isolated from the wounds. Student test was used and data were presented as means±SD. ***P < 0.001. (n = 3 in each group).

Original blot of Fig. 3B

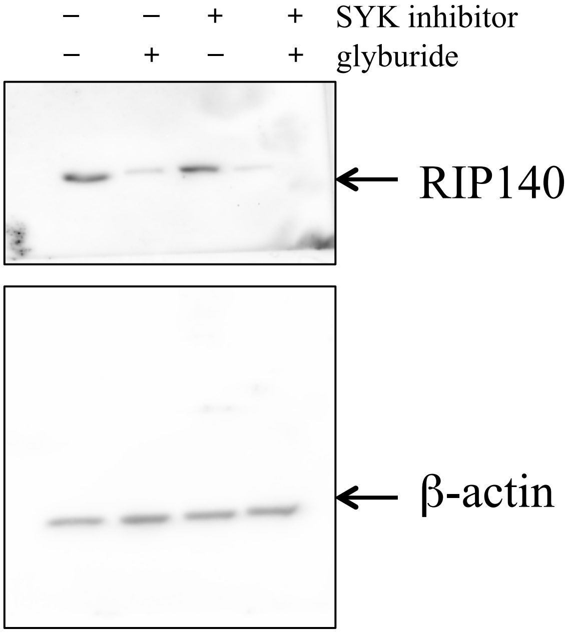

Original blot of Fig. 3C

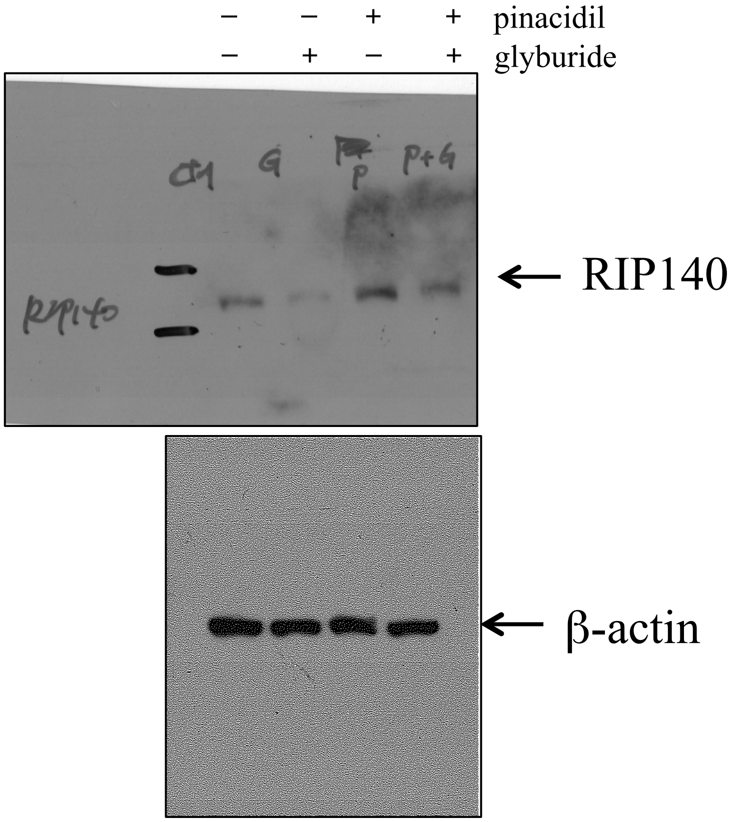

Original blot of Fig. 3D

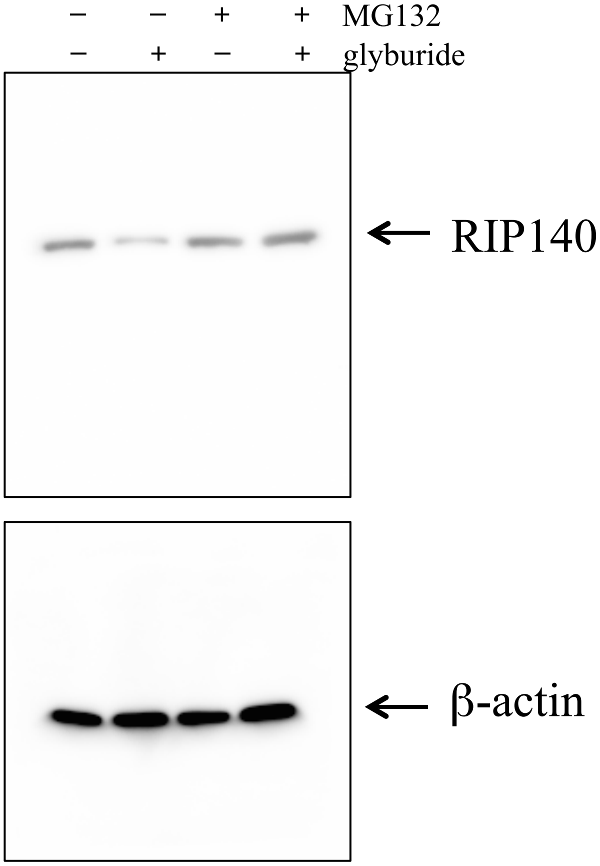

Original blot of Fig. 4A

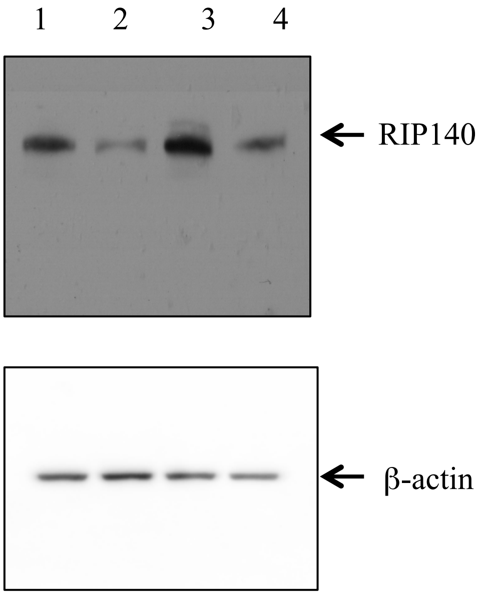

1: control
2: glyburide
3: glyburide+pan-CamKII inhibitor
4: BayK 8644

Original blot of Fig. 4B

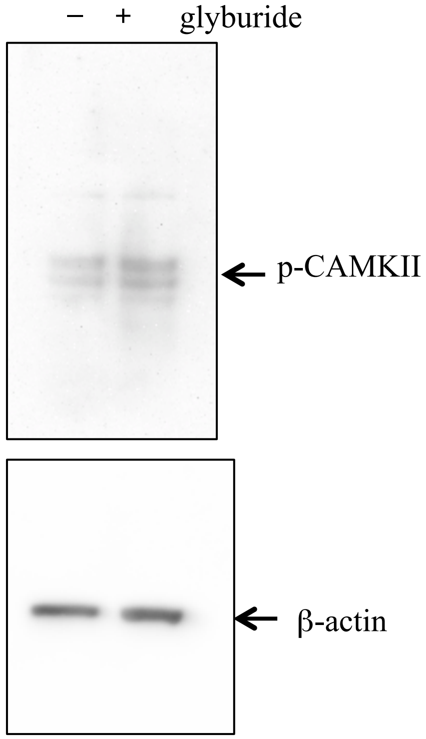

Original blot of Fig. 4C

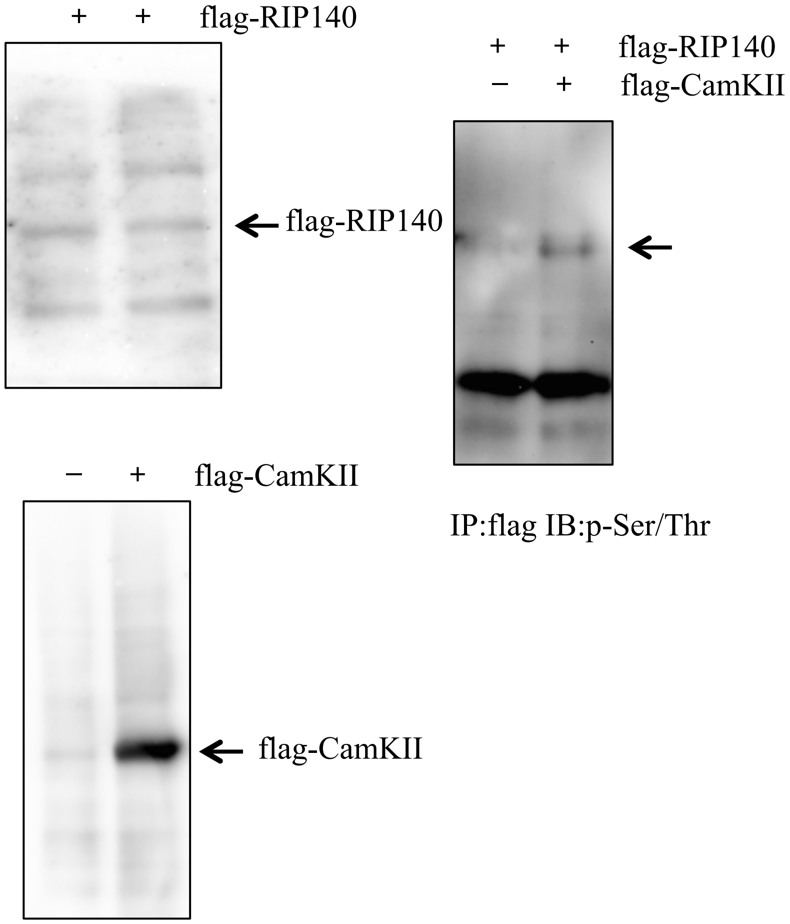

Original blot of fig. 4E

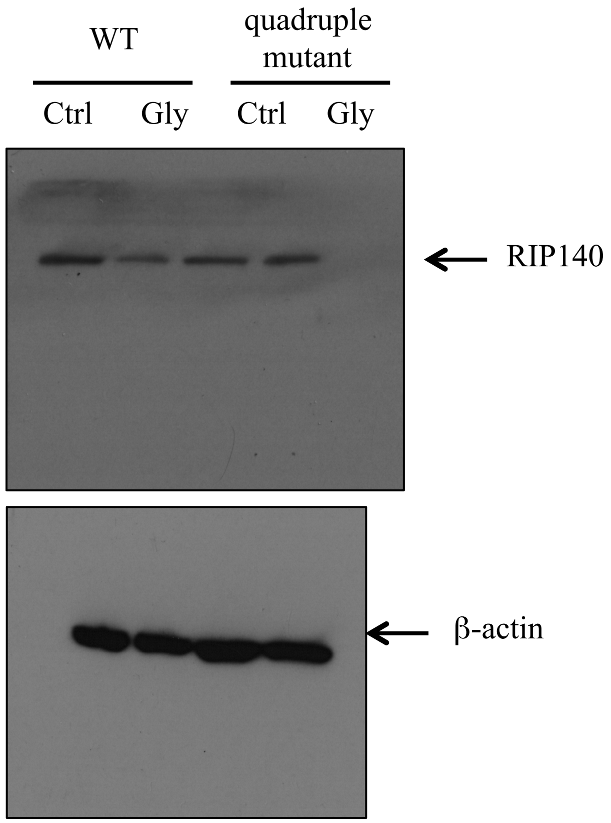

Supplement: Supplementary file 1 — Supplementary information [file 41598_2017_18785_MOESM1_ESM.pdf]
